# Radiomic features of amygdala nuclei and hippocampus subfields help to predict subthalamic deep brain stimulation motor outcomes for Parkinson‘s disease patients

**DOI:** 10.3389/fnins.2022.1028996

**Published:** 2022-10-13

**Authors:** Ausra Saudargiene, Andrius Radziunas, Justinas J. Dainauskas, Vytautas Kucinskas, Paulina Vaitkiene, Aiste Pranckeviciene, Ovidijus Laucius, Arimantas Tamasauskas, Vytenis Deltuva

**Affiliations:** ^1^Neuroscience Institute, Medical Academy, Lithuanian University of Health Sciences, Kaunas, Lithuania; ^2^Department of Neurosurgery, Medical Academy, Lithuanian University of Health Sciences, Kaunas, Lithuania; ^3^Department of Health Psychology, Faculty of Public Health, Medical Academy, Lithuanian University of Health Sciences, Kaunas, Lithuania; ^4^Department of Neurology, Medical Academy, Lithuanian University of Health Sciences, Kaunas, Lithuania

**Keywords:** Parkinson’s disease, deep brain stimulation, radiomic features, amygdala, hippocampus, motor outcome prediction

## Abstract

**Background and purpose:**

The aim of the study is to predict the subthalamic nucleus (STN) deep brain stimulation (DBS) outcomes for Parkinson’s disease (PD) patients using the radiomic features extracted from pre-operative magnetic resonance images (MRI).

**Methods:**

The study included 34 PD patients who underwent DBS implantation in the STN. Five patients (15%) showed poor DBS motor outcome. All together 9 amygdalar nuclei and 12 hippocampus subfields were segmented using Freesurfer 7.0 pipeline from pre-operative MRI images. Furthermore, PyRadiomics platform was used to extract 120 radiomic features for each nuclei and subfield resulting in 5,040 features. Minimum Redundancy Maximum Relevance (mRMR) feature selection method was employed to reduce the number of features to 20, and 8 machine learning methods (regularized binary logistic regression (LR), decision tree classifier (DT), linear discriminant analysis (LDA), naive Bayes classifier (NB), kernel support vector machine (SVM), deep feed-forward neural network (DNN), one-class support vector machine (OC-SVM), feed-forward neural network-based autoencoder for anomaly detection (DNN-A)) were applied to build the models for poor vs. good and very good STN-DBS motor outcome prediction.

**Results:**

The highest mean prediction accuracy was obtained using regularized LR (96.65 ± 7.24%, AUC 0.98 ± 0.06) and DNN (87.25 ± 14.80%, AUC 0.87 ± 0.18).

**Conclusion:**

The results show the potential power of the radiomic features extracted from hippocampus and amygdala MRI in the prediction of STN-DBS motor outcomes for PD patients.

## Introduction

During the last 30 years, deep brain stimulation (DBS) gained acceptance as a mainstream therapy for advanced Parkinson’s disease (PD) control. PD patients with an idiopathic disease course and responders to L-DOPA therapy are still treated as best candidates for deep brain stimulation (Pollak, 2013). Nevertheless, the clinical effect of this invasive therapy varies between PD patients, and better patient selection criteria or objective markers are needed.

It is considered that proper DBS electrode position within the subthalamic nucleus (STN) usually is a must for good clinical outcomes (Wodarg et al., 2012). Nevertheless, other factors play an essential role, and sometimes anatomically (Wodarg et al., 2012) and physiologically (Koirala et al., 2020) ideally implanted electrodes do not elicit satisfactory motor improvement after neuromodulation. Such inhomogeneous results between PD patients show challenges in proper patient selection for the therapy and the need for more accurate assessment tools.

Modern brain imaging techniques, detection of PD-associated radiogenomic features, and biochemical analysis of blood and cerebrospinal fluid empowers researchers for deeper disease understanding and specific clinical outcomes predicting markers discoveries (Hustad and Aasly, 2020). Resting-state functional magnetic resonance and diffusion tensor imaging for brain functional connectivity are explored as possible DBS outcomes predicting properties (Wang et al., 2021). Rapid development of open source and user-friendly radiomic tools also enables researchers to analyze patients’ radiological data from a different perspective.

For many decades degeneration of dopaminergic cells in the substantia nigra (SN) has been recognized as the primary locus for PD clinical symptoms (Damier et al., 1999; Colpan and Slavin, 2010). The recent growth of radiomics studies for different neurodegenerative brain disorders was also directed to PD and mostly to cortical and subcortical structures (Wu et al., 2019). Recently, it was also found that substantia nigra susceptibility features from radiomics could predict global motor and rigidity outcomes of STN-DBS in PD and suggested a predictive machine learning model for STN-DBS patient selection (Liu et al., 2021).

Alpha-synuclein is a presynaptic neuronal protein, and its structural alterations play an important role in the pathogenesis of neurodegenerative diseases, such as PD (Lücking and Brice, 2000). A recent study proposed a new model of PD pathogenesis with the alpha-synuclein origin site and connectome model, which divides PD into two subtypes: body-first subtype or brain-first subtype (Borghammer, 2021). According to this model, in the body-first subtype, alpha-synuclein pathology presumably originates in the enteric or autonomic nervous system and it spreads to the CNS, whereas the brain-first subtype alpha-synuclein pathology presumably originates in the amygdala or nearby structures. These different subtypes have different clinical onsets of PD. In the body-first subtype, the disease starts symmetrically, which is the opposite of the brain-first subtype. This model raises a hypothesis about different DBS outcomes for each PD subtype.

Significant advances have been achieved in the machine-learning (ML) based radiomic approach in recent years, aimed at improving the understanding of brain diseases and determining the most effective treatment options. Combining the extraction of radiomic features with ML methods can provide new non-invasive biomarkers for improved patient and disease characterization, and aid a better identification of DBS candidates (Liu et al., 2021; Ren et al., 2021). In the present study, we hypothesize that preoperative brain magnetic resonance image (MRI) radiomic analysis of the amygdalar-hippocampal region, one of the PD pathophysiological sites, may help differentiate patients who benefit most from the STN- DBS surgery. Radiomic features of amygdalar or nearby structures may serve as potentially new prognostic imaging biomarkers in planning PD patient treatment. The aim of our study is to develop the ML-based radiomics models to predict the STN-DBS motor outcome in PD patients.

## Materials and methods

### Study subjects

Adult patients diagnosed with PD were recruited in this prospective observational cohort study from Departments of Neurosurgery and Neurology of the Lithuanian University of Health Sciences Hospital, Kaunas, Lithuania. The study enrollment took place between April 2019 and August 2021.

The study inclusion criteria covered established diagnosis of L-DOPA responsive idiopathic PD, normal brain MRI scan results, the Mini-Mental State Examination (MMSE) score greater than 24 points, no active or untreated depression, no comorbid psychiatric disorders. L-DOPA responsiveness was assessed using the L-DOPA challenge test and was defined as 30% or greater improvement of the Unified Parkinson Disease Rating Scale (UPDRS) motor (III) part scores between off medication state and after administration of 1.5-fold higher than usual L-DOPA dose. The study exclusion criteria were atypical Parkinsonism, diagnosis of dementia or other current or past psychiatric disorders, and clinical comorbidities that precluded DBS implantation surgery. During the study period, 34 PD patients underwent the STN-DBS implantation surgery at our department. Five patients (15%, 4 males and 1 female) showed poor STN-DBS motor outcome, and the remaining 29 patients (85%, 12 males and 17 females) had good and very good motor effect of STN-DBS. The median age of the patients was 63 years (interquartile range 60-68 years) in the first group, and 60 years (interquartile range 57-62 years) in the second group. No significant differences of demographic characteristics were observed between the two groups (Table 1).

**TABLE 1:** Demographic data.

	Poor STN-DBS motor outcome	Good and very good STN-DBS motor outcome	Significance *p*
Number of patients	5	29	-
Age^a^	63 (60-68) years	60 (57-62) years	0.262
Gender^b^	Male: 4 (80%) Female: 1 (20%)	Male: 12 (41%) Female: 17 (59%)	0.164
PDCS preoperative^c^	18 (17-29)	22 (18-27)	0.609
PDCS postoperative^d^	18 (16-27)	11 (7-16)	0.034
LEDD preoperative^e^ (mg)	710 (620-840)	720 (620-800)	0.981
LEDD postoperative^f^ (mg)	710 (600-840)	300 (300-420)	<0.001
Displacement of the DBS electrode^g^, mm	0.2 (0.0-0.5)	0.3 (0.0-0.5)	0.603

Age, scores of the preoperative Parkinson’s Disease Composite Scale (PDCS) and postoperative PDCS, preoperative levodopa equivalent daily dose (LEED) and postoperative LEED (mg), DBS electrode displacement (mm) are presented as median and interquartiles (1st and 3rd). ^a^Kruskal-Wallis H test χ^2^(1) = 1.256; ^b^Fisher’s exact test statistics 5.667; ^c^Kruskal-Wallis H test χ^2^(1) = 0.262; ^d^Kruskal-Wallis H test χ^2^(1) = 4.491; ^e^Kruskal-Wallis H test χ^2^(1) = 0.001; ^f^Kruskal-Wallis H test χ^2^(1) = 11.662; ^g^Kruskal-Wallis H test χ^2^(1) = 0.271. STN-DBS – subthalamic nucleus deep brain stimulation; PDCS - Parkinson’s Disease Composite Scale; LEDD - levodopa equivalent daily dose.

Eligible PD patients were identified during routine clinical visits and invited to participate in the study. All PD patients preoperatively were evaluated for possible DBS surgery by a neurologist. According to CAPSIT-PD guidelines and a joint agreement between neurologists, neuropsychologist, and neurosurgeon patients were selected for DBS. All patients were operated on under general anesthesia following the same DBS electrode implantation protocol. Pre-implantation stereotactic MRI scans were acquired for safe lead guidance and proper targeting within STN. To maximize lead penetration within the motor part of STN, the target was chosen 2 mm from the medial border of STN at the maximal rubral diameter as described by Bejjani (Bejjani et al., 2000). Stereotactic postoperative MRI was used to confirm correct lead placement. The Parkinson’s Disease Composite Scale (PDCS) scores were calculated preoperatively and six months after DBS implantation. PDCS score improvement for more than 30% was considered a good or very good DBS outcome, and patients with lesser improvement were assigned to the poor DBS outcome group. The preoperative PDSC scores did not differ statistically significantly between the groups of poor vs. good and very good STN-DBS motor outcome (mean rank 15.40 vs. 17.86, respectively; *p* = 0.262; PDSC median 18 vs. 22), but the first group showed significantly higher postoperative PDSC scores (mean rank 26.20 vs. 16.00, respectively; *p* = 0.034; PDSC median 18 vs. 11) and higher doses of levodopa equivalent daily dose (LEDD) (mean rank 31.40 vs. 15.10, respectively; *p*<0.001; median 710 mg vs. 300 mg) (Table 1). The target for the DBS implantation was selected on a case-by-case basis by the neurosurgeon (AR).

After DBS electrode implantation, stereotactical MRI was performed for each patient. If the electrode position within STN deviated more than 1 mm from the primary target, the electrode would be re-implanted. None of this study patients needed reimplantation. Implanted electrode displacement, i.e., deviation from radiological target, was estimated for poor and good/very good outcome patients groups. The difference in displacement between the groups was not statistically significant (mean rank 15.40 vs. 17.86, respectively; *p* = 0.603; median 0.2 mm vs. 0.3 mm) (Table 1). These findings imply that clinical outcomes were not associated with the electrode position within STN.

### Data processing

**FIGURE 1:**
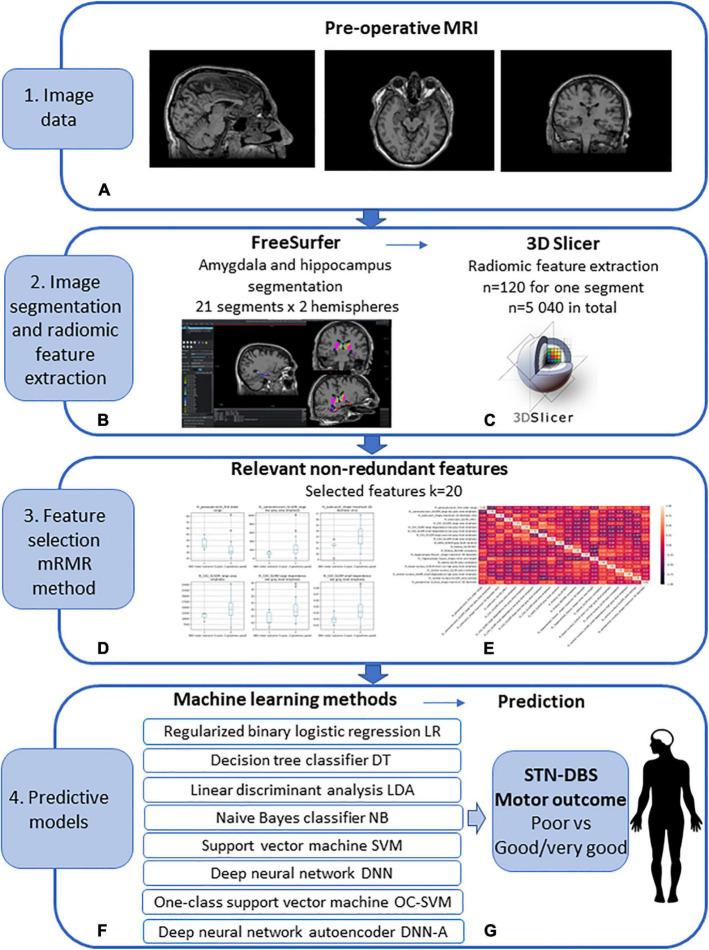
Workflow diagram of the pre-operative prediction of subthalamic nucleus (STN) deep brain stimulation (DBS) for Parkinson’s disease (PD) patients. (1) **(A)** Image data: pre-operative MRI image acquisition using the 1.5 T Siemens Avanto scanner. (2) Image segmentation and radiomic feature extraction: **(B)** amygdala and hippocampus segmentation using FreeSurfer; obtained 21 segments for 2 hemispheres; **(C)** radiomic feature extraction using 3D Slicer; extracted features *n* = 120 for every segment, n = 5 040 in total. (3) Feature selection using Minimum Redundancy Maximum Relevance (mRMR) method: **(D)** selected relevant features **(E)** selected non-redundant features *k* = 20. (4) Predictive machine learning models: **(F)** regularized binary logistic regression (LR), decision tree classifier (DT), linear discriminant analysis (LDA), naive Bayes classifier (NB), kernel support vector machine (SVM), deep feed-forward neural network (DNN), one-class support vector machine (OC-SVM), feed-forward neural network-based autoencoder for anomaly detection (DNN-A); **(G)** prediction of the STN-DBS motor outcome: poor vs. good/very good outcome.

The workflow diagram of the MRI image pre-processing, amygdala and hippocampus segmentation, radiomic feature extraction and selection, machine learning method application for post-operative prediction of STN-DBS motor outcome is shown in Figure 1.

#### Image pre-processing and segmentation

Preoperative T1W and T2W images were employed in FreeSurfer 7.0 module for automatic brain MRI morphometric data extraction and segmentation of hippocampal and amygdala subregions (Fischl, 2012; Sämann et al., 2022). The Freesurfer amygdalar-hippocampal pipeline segmented hippocampal-amygdala subregions simultaneously avoiding overlapping and producing 12 hippocampal subdivisions and 9 nuclei of the amygdala (Table 2). For each hemisphere, 21 segment were extracted, 42 segments in total (Figures 1A,B).

**TABLE 2:** Labeling of FreeSurfer’s amygdalar-hippocampal segmentation output and nuclei with selected radiomic features used to predict STN-DBS motor outcomes.

Nuclei/subfields			
**Amygdala**		**Hippocampus**	
***Nuclei***	***Radiomic feature***	***Subdivisions***	***Radiomic feature***

**Basal nucleus**	GLRLM short run high gray level emphasis	**Parasubiculum**	First order range, GLSZM large low gray area emphasis
**Lateral nucleus**	GLCM auto correlation	Presubiculum	-
Accessory basal nucleus	-	**Subiculum**	Shape maximum 2D diameter slice, GLCM IMC1
Anterior amygdaloid nucleus	-	**CA1**	GLSZM large area emphasis
**Central nucleus**	GLCM auto correlation, GLDM small dependence high gray level emphasis, GLSZM zone entropy	**CA3**	GLDM large dependence low gray level emphasis, GLDM small dependence low gray level emphasis, GLSZM large area low gray level emphasis
Medial nucleus	-	**CA4**	GLSZM small area emphasis
Cortical nucleus	-	GC-ML-DG	-
**Paralaminar nucleus**	Shape maximum 3D diameter	Molecular layer	-
Corticoamygdaloid	-	**HATA**	GLRLM gray level variance
Transition zone		**Fimbria**	GLCM MCC, NGTDM complexity
		Hippocampal tail	-
		**Hippocampal fissure**	Shape maximum 3D diameter, fissure shape minor axis length

GLRLM - Gray Level Run Length Matrix; GLSZM - Gray Level Size Zone Matrix; GLCM - Gray Level Co-occurrence Matrix; GLDM - Gray Level Dependence Matrix; MCC - Maximum Correlation Coefficient; NGTDM - Neighboring Gray Tone Difference Matrix; IMC1 - Informational Measure of Correlation 1; CA1-4 – Cornu Amonis; HATA - Hippocampal Amygdala Transition Area.

#### Radiomic feature extraction

The amygdalar-hippocampal FreeSurfer segmentation files were used in radiomic analysis for each patient. The radiomic feature extraction was performed by PyRadiomics, an open-source platform (van Griethuysen et al., 2017). A Radiomics extension for 3D Slicer was employed for the following radiomic feature extraction: 26 shape features, 19 first-order features, 24 Gray Level Co-occurrence Matrix (GLCM) features, 16 Gray Level Run Length Matrix (GLRLM) features, 16 Gray Level Size Zone Matrix (GLSZM) features, 14 Gray Level Dependence Matrix (GLDM) features, and 5 Neighboring Gray Tone Difference Matrix (NGTDM) features. The procedure described provided 120 radiomic features for each of 21 segments in both hemispheres, 5 040 features in total (Figure 1C).

#### Radiomic feature selection

The mRMR method (Zhao et al., 2019) was used for the extracted feature selection as it effectively reduces the redundant features while keeping the relevant features for the predictive ML models (Figures 1D,E). As the goal is to differentiate the patients into two classes of poor vs. good/very good STN-DBS motor outcome, the features that have maximum relevance with respect to the class prediction were determined using one-way ANOVA F-test. Feature redundancy was determined by Pearson correlation. The best subset of 20 features was formed by selecting the relevant features while controlling for the redundancy within the selected features. The number of features was chosen taking into account a small sample size of the PD patients (*N* = 34) and aiming to avoid overfitting. It is recommended that the sample-to-feature ratio should be at least close or higher than 2 (Raudys, 2001). Low sample-to-feature ratio results in ML model fitting the noise in the data and poor recognition accuracy when applied to unseen data (An et al., 2021).

#### Machine learning methods for STN-DBS motor outcome prediction

The selected informative features were applied to build models for the STN-DBS motor outcome prediction (Figures 1F,G). The following ML algorithms were applied: (1) regularized binary logistic regression (LR) with sigmoid function; the regularization term is equal to the sum of squares of all the feature weights; (2) decision tree classifier (DT); (3) linear discriminant analysis (LDA); (4) naive Bayes classifier (NB); (5) kernel support vector machine (SVM) with Gaussian radial basis function as a kernel function; (6) deep feed-forward neural network (DNN); the network had two hidden layers with 11 and 7 neurons with ReLU transfer functions, respectively, and one neuron with sigmoid activation function in the output layer; dropout technique was applied to randomly set a 10% fraction of visible and hidden neurons to zero during training to avoid overfitting, error-back propagation training algorithm with adaptive moment estimation was used; (7) one class support vector machine (OC-SVM) with Gaussian radial basis function as a kernel function; the negative output indicates the low density of observations and results in the detection of anomaly, i.e., the observation that deviates from the majority of the data significantly; poor STN-DBS outcome is regarded as anomaly; (8) deep feed-forward neural network-based autoencoder (DNN-A) for anomaly detection; the encoder consisted of input layer and one hidden layer with 18 neurons and ReLU transfer function, the latent layer had 4 neurons with ReLU transfer function, the encoder was formed of one hidden layer with 18 neurons and ReLU transfer function and output layer with 20 neurons and linear transfer function; dropout technique was applied to randomly set a 10% fraction of visible and hidden neurons to zero; error-back propagation training algorithm with adaptive moment estimation was used; the anomaly was identified if the error of the reconstructed 20-dimensional feature vector was higher by one standard deviation than the mean square error of the reconstructed samples from the class of good and very good DBS motor outcome.

The available data set (*N* = 34 patients) was split into training and testing sets. The training set consisted of 85% of all data set (4 cases from the class of poor STN-DBS motor outcome and 24 cases from the class of good/very good STN-DBS motor outcome), and the remaining 15% of the data were used for testing (1 case and 5 cases from each class, respectively). Prior to the ML analysis training and testing sets were standardized using a standard scaler. Training and testing data were bootstrapped with 1,000 repetitions, drawing a sample data repeatedly with replacement from the available data set. To balance the unequal size of datasets, the class weights were estimated and used in machine learning algorithms. The accuracy, specificity, sensitivity, and area of the receiver operating characteristic (ROC) curve (AUC) of the ML models were estimated and averaged over 1,000 bootstrapped repetitions.

Radiomic feature selection, data processing, and machine learning method implementation were performed using sci-kit-learn Python package and Keras Python framework (Pedregosa et al., 2011).

## Results

The segmentation of amygdala and hippocampus regions, radiomic feature extraction and selection resulted in defining the 20 most informative features that represent the amygdala basal nucleus, lateral nucleus, central nucleus, paralimar nucleus, and the hippocampal parasubiculum, subiculum, CA1, CA3, CA4, HATA, fimbria, hippocampal fissure.

The following 20 features were selected out of the extracted 5 040 features using the mRMR method: *amygdala*: lh_lateral_GLCM auto correlation, lh_basal nucleus_GLRLM short run high gray level emphasis, lh_central nucleus_GLCM auto correlation, lh_central nucleus_GLDM_small dependence high gray level emphasis, lh_central nucleus GLSZM_zone entropy, lh_paralaminar nucleus_shape maximum 3D diameter; *hipoccampus:* lh_parasubiculum_first order range, lh_ parasubiculum_GLSZM_large low gray area emphasis, lh_subiculum_shape maximum 2D diameter slice, lh_subiculum_GLCM_IMC1, lh_CA1_GLSZM_large area emphasis, lh_CA3_GLMD large dependence low gray level emphasis, lh_CA3_GLDM small dependence low gray level emphasis, lh_CA3_GLSZM large area low gray level emphasis, lh_CA4_GLSZM small area emphasis, Ih_HATA_GLRLM gray level variance, Ih_fimbria_GLCM MCC, lh_fimbria_NGTDN complexity, lh_hippocampal_fissure _shape maximum 3D diameter, lh_ hippocampal_fissure_shape minor axis length.

**FIGURE 2:**
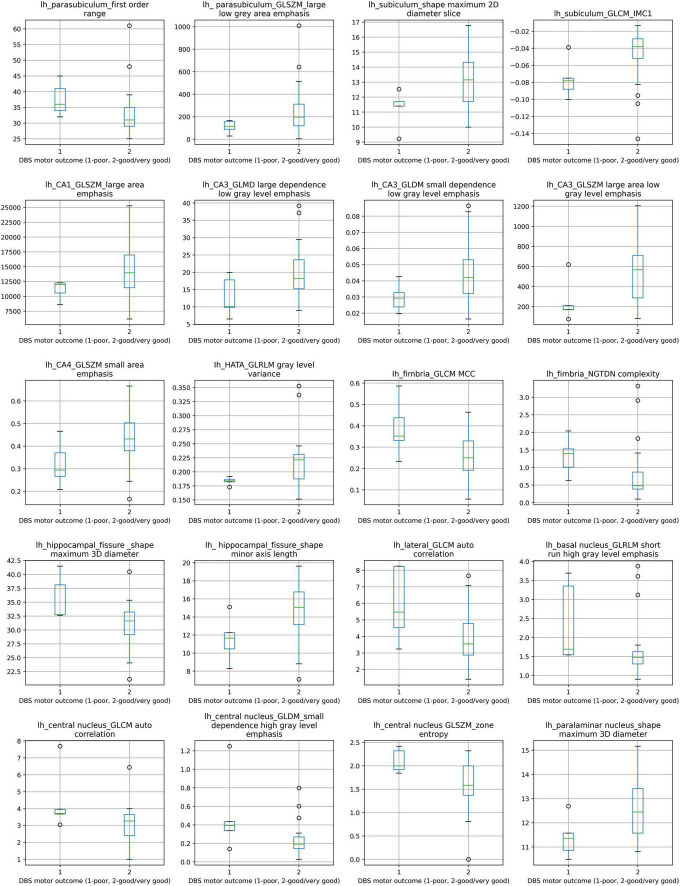
Box plots of comparison between poor STN-DBS outcome and good/very good STN-DBS outcome for 20 selected radiomic features. Distributions of features between two STN-DBS groups differ statistically significantly, Kruskal-Wallis H test χ^2^(1) and *p* value (in parentheses): lh_parasubiculum_first order range: 4.402 (0.036), lh_ parasubiculum_GLSZM_large low gray area emphasis: 3.878 (0.049), lh_subiculum_shape maximum 2D diameter slice: 3.980 (0.046), lh_subiculum_GLCM_IMC1: 4.682 (0.030), lh_CA1_GLSZM_large area emphasis: 3.878 (0.049), lh_CA3_GLMD large dependence low gray level emphasis: 3.878 (0.049), lh_CA3_GLDM small dependence low gray level emphasis: 4.271 (0.039), lh_CA3_GLSZM large area low gray level emphasis: 3.878 (0.049), lh_CA4_GLSZM small area emphasis: 3.878 (0.049), Ih_HATA_GLRLM gray level variance: 3.878 (0.049), Ih_fimbria_GLCM MCC: 3.878 (0.049), lh_fimbria_NGTDN complexity: 5.113 (0.024), lh_hippocampal_fissure _shape maximum 3D diameter: 4.274 (0.039), lh_ hippocampal_fissure_shape minor axis length: 4.474 (0.034), lh_lateral_GLCM auto correlation: 3.878 (0.049), lh_basal nucleus_GLRLM short run high gray level emphasis: 3.878 (0.049), lh_central nucleus_GLCM auto correlation: 3.879 (0.049), lh_central nucleus_GLDM_small dependence high gray level emphasis: 3.878 (0.049), lh_central nucleus GLSZM_zone entropy: 4.331 (0.037), lh_paralaminar nucleus_shape maximum 3D diameter: 5.005 (0.025). Notation *lh* at the feature label indicates left hemisphere.

**FIGURE 3:**
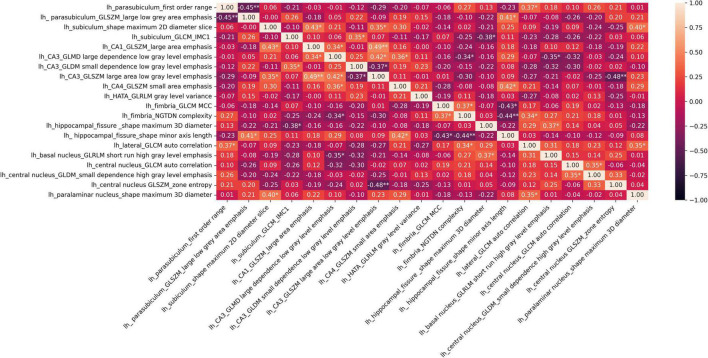
Heatmap of Spearman‘s rho correlation coefficients of 20 selected radiomic features; *p < 0.05, ^**^p < 0.001 show significant associations.

The distributions of the 20 selected radiomic features in the classes of poor vs. good/very good STN-DBS motor outcomes are presented in box plots (Figure 2). The box plots clearly display the deviation in the medians of the features in these two groups. The differences in distributions are statistically significant for all features (Kruskal Wallis H test *p* < 0.05) and confirm the maximum relevance with respect to the motor outcome prediction. Spearman‘s rho correlation coefficients between the features vary from −0.48 to + 0.49 and show moderate, weak or no association ensuring the minimum redundancy (Figure 3).

**FIGURE 4:**
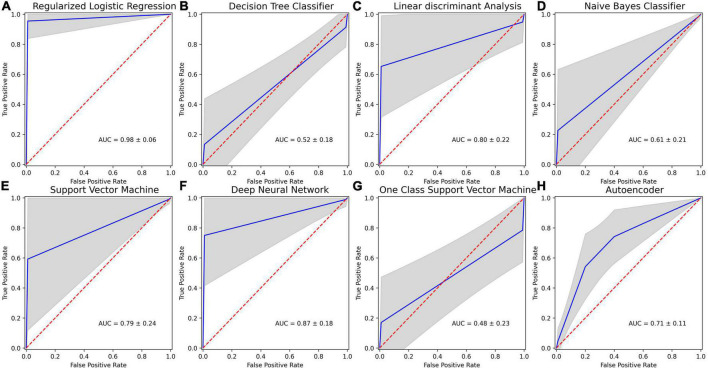
Receiver operating characteristics (ROC) curves for machine learning (ML) methods applied in post-operative STN-DBS motor outcome prediction using 20 selected features: **(A)** regularized binary logistic regression (LR); **(B)** decision tree classifier (DT); **(C)** linear discriminant analysis (LDA); **(D)** naive Bayes classifier (NB); **(E)** kernel support vector machine (SVM); **(F)** deep feed-forward neural network (DNN); **(G)** one-class support vector machine (OC-SVM); **(H)** feed-forward neural network-based autoencoder for anomaly detection (DNN-A). Mean ROC is shown in blue, and one standard deviation is represented by the gray area. Dotted red line indicates chance performance, and curves that deviate more to the top left represent better predictions.

The selected features were used to predict poor STN-DBS motor outcome vs. good/very good effect, i.e., to identify the complications in the post-operative STN-DBS motor functioning. The accuracy, specificity, sensitivity and AUC of the machine learning algorithms are presented in Table 3. The ROC curves together with AUC of the models are shown in Figure 4.

**TABLE 3:** Accuracy, sensitivity, specificity and AUC of the ML methods applied in post-operative STN-DBS motor outcome prediction using 20 selected features.

No.	Machine learning method	Accuracy %	Sensitivity %	Specificity %	Area under ROC (AUC)
1.	**Regularized binary logistic regression (LR)**	**96.65 ±** 7.24	**99.30 ±** 8.34	**96.12 ±** 8.59	**0.98 ±** 0.06
2.	Decision tree classifier (DT)	77.42 ± 12.47	14.60 **±** 35.33	89.98 **±** 14.15	0.52 ± 0.18
3.	Linear discriminant analysis (LDA)	77.90 ± 18.28	83.20 ± 37.41	76.84 ± 19.64	0.80 ± 0.22
4.	Naive Bayes classifier (NB)	86.62 ± 7.27	21.90 ± 41.38	99.56 ± 3.07	0.61 ± 0.21
5.	Support vector machine (SVM)	91.72 ± 9.04	60.40 ± 48.93	97.98 ± 6.03	0.79 ± 0.24
6.	**Deep feed-forward neural network (**DNN**)**	**87.25** ± 14.80	**86.60** ± 34.08	**87.38** ± 17.48	**0.87** ± 0.18
7.	One class support vector machine (OC-SVM)	63.33 ± 17.88	24.40 ± 42.97	71.12 ± 20.65	0.48 ± 0.23
8.	Deep neural network autoencoder (DNN-A)	70.73 ± 10.68	73.36 ± 11.60	68.10 ± 22.91	0.71 ± 0.11

The bold values indicate the highest accuracy.

Low sensitivity and low AUC in identifying poor STN-DBS outcome were obtained using DT classifier (accuracy 77.42 ± 12.47%, sensitivity 14.60 **±** 35.33%, specificity 89.98 **±** 14.15%, AUC 0.52 ± 0.18), NB classifier (accuracy 86.62 ± 7.27%, sensitivity 21.90 ± 41.38%, specificity 99.56 ± 3.07%, AUC 0.61 ± 0.21), OC-SVM (accuracy 63.33 ± 17.88%, sensitivity 24.40 ± 42.97%, specificity 71.12 ± 20.65%, AUC 0.48 ± 0.23). DT tree classifier is based on the hierarchical sequence of decisions on the features and is sensitive to small variations in the training set, especially for such complex data in high dimensional feature space as radiomics measures. NB classifier assumes that features are independent and drawn from Gaussian distribution, which is not the case in our study. OC-SVM identifies the anomalies (poor STN-DBS outcome) as data outside the learned decision boundary of normal cases (good/very good STN-DBS outcome). OC-SVM can form complex boundaries in high dimensional feature space and is less prone to overfitting resulting in poor accuracy.

Moderately good performance was obtained by LDA (accuracy 77.90 ± 18.28%, sensitivity 83.20 ± 37.41%, specificity 76.84 ± 19.64%, AUC 0.80 ± 0.22), DNN-A (accuracy 70.73 ± 10.68%, sensitivity 73.36 ± 11.60%, specificity 68.10 ± 22.91%, AUC 0.71 ± 0.11), and SVM (accuracy 91.72 ± 9.04%, sensitivity 60.40 ± 48.93%, specificity 97.98 ± 6.03%, AUC 0.79 ± 0.24). LDA fails to discriminate non-linearly separable classes and gives very high variability of sensitivity due to heterogeneity of data. DNN-A showed good sensitivity, i.e., the reconstruction error of the presented poor STN-DBS outcome data was high indicating that the sample was an anomaly. However, due to a complex DNN-A architecture a moderate proportion of good and very good STN-DBS samples were also reconstructed with high errors, resulting in reduced specificity. SVM searches for the linear optimal separating hyperplane in the transformed feature space using so called support vectors, i.e., points closest to the hyperplane from both classes. In our study, this method suffers from an imbalanced data set and favors a class with a large number of samples. As the class of good/very good STN-DBS outcomes is larger in this study, SVM gives high specificity and a low sensitivity.

The highest overall prediction accuracy scores were achieved by regularized binary LR (accuracy 96.65 ± 7.24%, sensitivity 99.30 ± 8.34%, specificity 96.12 ± 8.59%, AUC 0.98 ± 0.06) and DNN (accuracy 87.25 ± 14.80%, sensitivity 86.60 ± 34.08%, specificity 87.38 ± 17.48%, AUC 0.87 ± 0.18). Binary LR required a few parameters to be estimated and was regularized to avoid overfitting, therefore resulting in high sensitivity and specificity with the low variance. Although DNN performance has large variability in sensitivity due to the deep neural network architecture and limited sample size, the achieved accuracy is promising and shows the potential of DNN application in identifying poor STN-DBS outcomes.

In ML imbalanced datasets lead to high majority baselines as we have in our study (majority baseline = 85.29%). LR and DNN exceed this threshold, show high sensitivity and specificity, and therefore these methods can be considered suitable for solving the defined problem.

The results indicate that the task of STN-DBS outcome prediction is complex, and involves multiple steps as radiomic feature extraction from the MRI images, feature selection, ML model development and training, and accuracy evaluation. The high dimensional feature space and limited sample size require achieving a trade-off between ML method complexity and its performance.

## Discussion

The primary aim of this study was to investigate the potential of radiomic features of amygdalar nuclei and hippocampus subfields to predict the STN-DBS motor outcomes for PD patients. The selected features used in machine learning algorithms provide high prediction accuracy, sensitivity, specificity and AUC.

To select the best candidates for DBS surgery the CAPSIT-PD guidelines (Defer et al., 1999) were strictly followed. Despite this, only 1.6% of PD subjects would be eligible for DBS (Morgante et al., 2007). Moreover, the last decades of extensive research added new neuropsychological (Pal et al., 2015), radiological (Wang et al., 2021), and genetic (Artusi et al., 2019; de Oliveira et al., 2019) markers to achieve the best clinical outcomes. Furthermore, functional MRI data-driven studies showed increased overall connectivity in the motor network with strengthening thalamo-cortical connectivity in PD patients after DBS implantation (Abboud et al., 2015), that drives new discussions on preoperative functional brain connectome importance for best candidates’ selection. Furthermore, it is known that DBS clinical outcomes are associated with volumes of tissue activated and tracts stimulated (Lai et al., 2021). Nevertheless, an abundance of suggested radiological tools and hypotheses for the selection of best candidates for surgery drives new data-driven markers.

The SN and STN structural changes are considered as main factors for clinical deterioration in patients with advanced PD (Damier et al., 1999; Colpan and Slavin, 2010). SN radiomic features have been employed in ML algorithms to discriminate between patients with PD and healthy subjects (Li et al., 2019; Xiao et al., 2019; Xiao et al., 2021). The recent pilot study (Liu et al., 2021) demonstrated that SN radiomic features combined with the binary logistic regression analyses could predict motor outcome of STN-DBS in PD with 82% accuracy (AUC = 0.85).

Usually, studies of DBS outcome prediction include analysis of connectivity profiles (Horn et al., 2017; Lai et al., 2022), functional magnetic resonance imaging fMRI data (Boutet et al., 2021), local field potentials (Wang et al., 2018), EEG analysis (Geraedts et al., 2021), clinical performance scores (Habets et al., 2019).

Horn et al., 2017 showed that structural and functional connectivity is associated with STN-DBS outcome in PD giving medium correlation *R* = 0.51, *p* < 0.001. General linear model predicted UPDRS-III improvements with 15.7 ± 14.2% deviation from the actual value in single patients. The results suggested that functional connectivity adds predictive value above and beyond anatomical connectivity. The study by Lai et al. (2022) indicates that functional connectivity patterns predicted globus pallidus internal (GPi) DBS outcome with the deviation of 13.1% ± 11.3% and correlation *R* = 0.58 (*p* = 0.006) from the actual improvements. Boutet et al. (2021) investigated fMRI responses to DBS stimulation to predict optimal stimulation settings for individual patients. LDA model achieved 88% training accuracy in classifying optimal versus non-optimal parameter settings. Bermudez et al. (2019) used MRI data and convolutional neural network to classify a DBS electrode coordinate as having a positive or negative response to stimulation. This method achieved an AUC of 0.627 with a sensitivity of 0.338 and specificity of 0.849.

Recent review shows that modern ML algorithms such as DNN, convolutional neural networks, recurrent neural networks, long-short term memory neural networks alongside the conventional algorithms such as LR, LDA, SVM, are increasingly being used to analyze imaging, local field potentials, EEG, microelectrode recordings (MER) data in DBS research. However, none of 73 studies employed radiomic features (Peralta et al., 2021).

To our knowledge, our study is the first study to investigate the radiomic features extracted from thalamic and amygdalar-hippocampal nuclei as predictors of the STN-DBS motor effect. Our results show that ML algorithms, merged with the selected features, lead to 96.7% accuracy (AUC = 0.98) and indicate the importance of amygdalar-hippocampal region in PD disease treatment outcome prediction.

It is known, that the amygdala and hippocampus are important structures for animal and human cognition and serve as a crucial hub for cortical, subcortical, and limbic connections throughout the brain (Churchyard and Lees, 1997; Izquierdo et al., 1997; Adolphs, 2010). Until now pathological changes in this region are usually considered as markers for possible cognitive impairment in different neurological disorders. But new PD models with possible two PD types according to alpha-synuclein deposits in different body parts put the amygdala into a new role in PD pathophysiology (Borghammer, 2021). Our results elicit the importance of the amygdala and hippocampus role in PD patient motor functioning after STN-DBS. This region should be explored extensively using other tools to bring strong evidence about possible reasons for founded radiomic changes.

Radiomic is based on statistical-based modeling, which is usually employed to extract many quantitative features from MRI using data characterization algorithms and giving semantic and agnostic features as outputs (Forghani, 2020). Misfolded alpha-synuclein or Lewy body itself, on its crucial role in PD pathophysiology, might be a candidate for possible radiomic changes in amygdala and hippocampus in our study. Per Borghammers’ suggested PD model supports this assumption because brain origin PD type might be considered as a classical idiopathic PD with good L_DOPA therapy response and which is also suitable for DBS according to its clinical course and features. Five patients from our study with poor clinical outcomes, despite of similar clinical state, received DBS at the later PD stage when alpha-synuclein in the amygdala decreased, which led to poorer STN DBS results.

Evidence from human post-mortem studies suggests that alpha-synuclein pathology could spread from the enteric nervous system and propagate to the central nervous system (CNS) through the vagal nerve (Braak et al., 2003). As Braak proposed, alpha-synuclein spreading routes to SN through hindbrain, basolateral amygdala, dorsal raphe nucleus. An experimental animal study of transneuronal propagation of pathologic alpha-synuclein (Kim et al., 2019) showed it deposits in amygdala, which corresponds with Braak 1 stage hypothesis. Our results also strongly suggested amygdala importance in STN - DBS outcomes. We presume that radiomic changes in amygdala show pathologic alpha-synuclein. Amygdala at this stage of PD has a higher amount of alpha-synuclein than SN. This observation drives the following hypothesis: the PD patients are better candidates for STN-DBS if the disease stage show alpha-synuclein deposits still at the level of amygdala. Five patients of our study with poor clinical outcomes, despite of similar clinical state, received DBS at the later PD stage when alpha-synuclein in the amygdala decreased, which led to poorer STN DBS results.

The limitation of this work is the small number of PD patients included in the study. The conclusions on the prediction accuracy of the DBS effect for PD patients remain to be fully validated using a larger dataset.

## Conclusion

The obtained results show the potential of hippocampus and amygdala radiomic features in the prediction of STN-DBS motor outcomes for PD patients. The amygdala and hippocampus radiomic changes should be explored on a bigger scale for their importance in PD patient selection for STN-DBS. Radiomic features at the level of amygdala might show pathological deposits of alpha-synuclein. Nevertheless, correlation of radiomic features with immunohistochemical PD patient data is crucial for our hypothesis validation.

## Data availability statement

The original contributions presented in this study are included in the article, further inquiries can be directed to the corresponding author.

## Ethics statement

The studies involving human participants were reviewed and approved by Ethics Committee for Kaunas Region Biomedical Research at the Lithuanian University of Health Sciences, Kaunas, Lithuania. The patients/participants provided their written informed consent to participate in this study.

## Author contributions

AR: conception, radiomic feature extraction, and writing the first draft. AS: statistical data analysis and machine learning methods, writing methods, results section, and discussion. JJD and VK: statistical data analysis and machine learning methods. OL: neurological patient assessment and critical revision. VK, AP, VD, and AT: review and critical revision. All authors read and approved the final manuscript.
